# Needle-Shaped Biosensors for Precision Diagnoses: From Benchtop Development to In Vitro and In Vivo Applications

**DOI:** 10.3390/bios14080391

**Published:** 2024-08-13

**Authors:** Ruier Xue, Fei Deng, Tianruo Guo, Alexander Epps, Nigel H. Lovell, Mohit N. Shivdasani

**Affiliations:** 1 Graduate School of Biomedical Engineering, UNSW Sydney, Sydney, NSW 2052, Australia; sherie.xue@student.unsw.edu.au (R.X.); fei.deng@unsw.edu.au (F.D.); t.guo@unsw.edu.au (T.G.); a.epps@student.unsw.edu.au (A.E.); n.lovell@unsw.edu.au (N.H.L.); 2 Tyree Foundation Institute of Health Engineering (IHealthE), UNSW Sydney, Sydney, NSW 2052, Australia

**Keywords:** needle-shaped biosensor, in situ detection, precision diagnosis, complex clinical samples, clinical biomarkers

## Abstract

To achieve the accurate recognition of biomarkers or pathological characteristics within tissues or cells, in situ detection using biosensor technology offers crucial insights into the nature, stage, and progression of diseases, paving the way for enhanced precision in diagnostic approaches and treatment strategies. The implementation of needle-shaped biosensors (N-biosensors) presents a highly promising method for conducting in situ measurements of clinical biomarkers in various organs, such as in the brain or spinal cord. Previous studies have highlighted the excellent performance of different N-biosensor designs in detecting biomarkers from clinical samples in vitro. Recent preclinical in vivo studies have also shown significant progress in the clinical translation of N-biosensor technology for in situ biomarker detection, enabling highly accurate diagnoses for cancer, diabetes, and infectious diseases. This article begins with an overview of current state-of-the-art benchtop N-biosensor designs, discusses their preclinical applications for sensitive diagnoses, and concludes by exploring the challenges and potential avenues for next-generation N-biosensor technology.

## 1. Introduction

Biomarker tests such as blood tests [1], urinalysis [2], stool examination [3], and genetic testing [4] provide objective indicators for precise diagnoses of diseases. These tests offer valuable insights into an individual’s health, and they are essential for accurate and tailored medical assessments. However, these tests usually require relatively large sample volumes (exceeding 50 μL) and involve long detection times, as well as labour-intensive sample-processing steps. These factors are particularly evident when testing clinical analytes derived from complex biological samples like blood [5], saliva [6], or stool [7]. There is a significant unmet clinical need for new approaches that can be conducted directly at the point-of-care (POC) for the in situ monitoring of biomarkers, eliminating the need for tedious clinical sample collection and processing.

Needle-shaped biosensors (N-biosensors) comprise a category of biosensors constructed on the surface of deployable needle-shaped substrates. These substrates can include optical fibres [8], stainless steel wires [9], and microneedles [10]. N-biosensors have been used to detect a range of biomarkers in vitro from blood [11], cerebrospinal fluid [9], and biopsied lung tissue [12]. Unlike conventional plate-based detection approaches (e.g., ELISA), which involve placing all clinical samples directly into a detection system, an N-biosensor can capture target biomarkers from complex clinical biospecimens in situ. Additionally, the in vivo sampling of clinical biospecimens is typically accompanied by higher surgical complications, and it requires a more precise approach to diagnosis. An N-biosensor offers a practical means for the in vivo detection of biomarkers whilst mitigating such surgical risks, achieved by inserting the biosensor directly into the target organ or tissue of interest. For instance, custom-designed N-biosensors have been successfully implemented for in vivo measurements of cytokines from the rat intrathecal space and discrete brain regions [9,13]. Therefore, N-biosensors have substantial potential for applications in biomarker detection both in vitro and in vivo.

**Figure 1:**
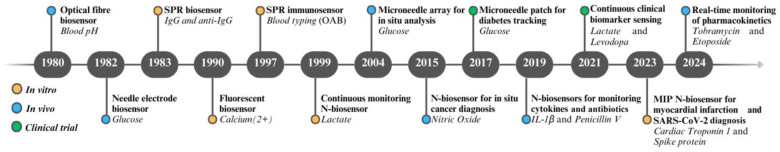
Timeline highlighting significant milestones in the development and application of needle-shaped biosensors (N-biosensors) over the past few decades. These landmark biosensors include optical fibre sensor for measuring blood PH [14], needle electrode sensor for monitoring glucose [15], SPR sensor for detecting IgG and anti-IgG [16], fluorescent sensor for measuring Calcium ions [17], SPR sensor for identifying blood type [5], sensor for continuously monitoring lactate [18], microneedle array for analysing glucose [19], sensor for detecting Nitric Oxide [20], microneedle sensor for tracking glucose dynamics [21], sensor for detecting IL-1β [8,9] and Penicillin V [22], sensors for continuously monitoring Lactate [23] and levodopa [24], MIP sensor for cardiac troponin 1 [25] and spike protein [26], and sensor for real-time monitoring Tobramycin [27] and Etoposide [28].

The development of N-biosensors has progressed significantly over the past few decades. Figure 1 demonstrates a timeline of key developments in N-biosensor technology, highlighting significant milestones in their design and application. Initially using optical fibres and needle-shaped electrodes at inception, the technology has since advanced to incorporate microneedles and other innovative substrates. Evolutions in this technology have further enabled the detection of a broader range of biomarkers with increasing precision and sensitivity.

In this review, we focus on the development and application of N-biosensors. We first describe the benchtop development of N-biosensors, discussing the design of each component, with a particular emphasis on the immobilisation approaches to the recognition module as a critical element of sensor development. We then provide a broad overview of the diverse diagnostic applications of both in vitro and in vivo N-biosensors. We offer insights into how these tools simplify sample collection and enable the minimally invasive in vivo detection of disease biomarkers, facilitating precision diagnoses. Finally, we present an outlook into next-generation N-biosensors that address current challenges in sensor design, and then we highlight potential enhancements in clinical performance and emphasise the role of sensor multiplexing and real-time in vivo monitoring in future N-biosensor development.

## 2. The Design of N-Biosensors

**Figure 2:**
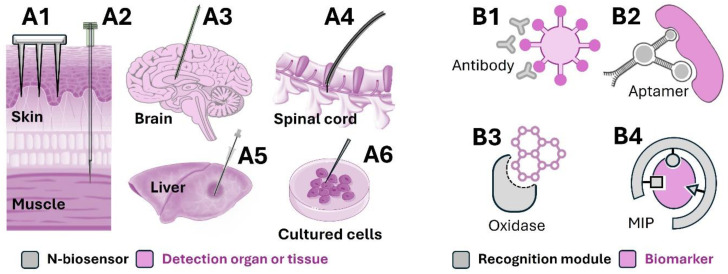
Examples of clinically used N-biosensor designs detailing their physical structures, recognition modules, and targeted organs or tissues. Commonly employed N-biosensor structures include microneedles (**A1**), acupuncture needles (**A2**), stainless steel needles (**A3**–**A5**), and nanoneedles (**A6**). The choice of N-biosensor shape also depends on the targeted organ or tissue. The primary recognition modules utilised are antibodies (**B1**), aptamers (**B2**), oxidases (**B3**), and MIPs (**B4**).

The comprehensive benchtop design of N-biosensors involves three main steps, mirroring the fundamental process for general sensor design. Step 1 entails selecting suitable materials for each component, including the supporting substrate, functional material, recognition module, and signal transducer. Step 2 focuses on choosing the optimal approach to immobilising the recognition module onto the functionalised substrate. Step 3 integrates all components into a fully functional N-biosensor. Table 1 highlights various N-biosensors, including details on their designs employed to detect biomarkers for various targeted applications. Examples of different clinically used N-biosensor designs can be found in Figure 2.

### 2.1. Options for N-Biosensor Components

#### 2.1.1. Supporting Substrate and Functional Material

The supporting substrate serves as the structural foundation of the N-biosensor and provides a basis for immobilising functional materials. Ideal candidates for supporting substrates are those with superior mechanical properties and chemical stability, such as optical fibres [29], stainless steel wires [11,31], and polymers [37]. Advanced functional materials, such as polymers [12] or nanoparticles [44], can significantly enhance N-biosensing capabilities by providing the superior immobilisation of recognition modules [8], the amplification of transduction signals [44], and the reduction in nonspecific adsorption [11]. Routine methods for attaching functional materials to the surface of a supporting substrate include physical adsorption [42] or chemical synthesis [11].

The choice of supporting substrate and functional material for an N-biosensor largely depends on the type of signal transduction method, including fluorescence, colourimetric, or electrochemical signalling. Fluorescent N-biosensing is a common approach to the in situ detection of diverse biomarkers [9,11,45]. The supporting substrates of a fluorescent N-biosensor require high mechanical strength, like stainless steel [11] and optical fibres [8], to withstand the force of insertion into tissue. Corresponding functional materials provide a range of specific properties. For instance, streptavidin is frequently used to immobilise biotinylated recognition domains due to its high binding affinity to biotin [8]. Gold film may be applied as a functional material to amplify the transducer signal through surface-enhanced fluorescence technology [46]. Additionally, polymer materials can provide antifouling properties to reduce nonspecific adsorption [11]. Electrochemical N-biosensing is a promising platform for the in situ testing of clinical analytes. To develop an electrochemical N-biosensor, diverse needle-shaped conductive materials have been utilised as the supporting substrate, including gold [31], carbon [35], and graphite [42]. These substrates frequently undergo modification with conductor materials based on nanoparticles or films for signal amplification, such as gold films [33], gold nanoparticles [35], and conductive polymers [39].

#### 2.1.2. Recognition Module and Signal Transducer

The selection of a suitable recognition module depends on the nature of the target biomarkers. As shown in Table 1, biomarkers detected through N-biosensing are classified into five categories: (1) small proteins, (2) small molecules, (3) nucleic acids such as deoxyribonucleic acid (DNA), (4) microorganisms, and (5) cells.

Small proteins, such as insulin, can be detected using various recognition modules, including antibodies, aptamers, molecularly imprinted polymers (MIPs), affibodies, corona phase polymers, and bioreceptors [47]. The choice among these types for the same target is primarily based on binding affinity and selectivity.

The detection of small molecules, such as glucose, lactate, and oxalate, primarily relies on bioreceptor, enzyme, aptamer, or MIP techniques for recognition [34,35,41]. Bioreceptors are the most commonplace among these options, constituting the only naturally occurring recognition modules produced by living organisms [35]. Enzyme-based N-biosensing offers specific advantages, as, in addition to its recognition ability for the target molecule, it also has the capability of signal amplification due to its enzymatic catalytic properties [37,41]. Aptamer approaches have increasingly been reported, enabling the recognition of small molecule biomarkers without size and antigenicity restrictions [34]. For the MIP approach, a biomimetic recognition module of over-oxidised polypyrrole has been used, emulating the target molecule as a template [39]. This approach offers exceptional physical and chemical stability compared to conventional protein-based recognition modules (e.g., antibodies, enzymes, and bioreceptors) [30].

The recognition of DNA targets can be achieved either using the target’s complementary DNA or through MIP approaches. Complementary DNA is the natural recognition module for DNA targets, and it exhibits high affinity [42]. However, when detecting DNA targets in complex biological samples, an MIP-based N-biosensor may be preferable due to its superior chemical stability [47].

Microorganism sensing in analytes for clinical diagnosis includes *E. coli* [48], *S. typhimurium* [49], and *V. cholerae* [50]. This process incorporates recognition modules such as phage [48], antibody [50], antimicrobial peptide [49], and siderophore components [51]. The final biomarker class is cells, where antibody-based or aptamer-based sensors are employed to recognise cell-surface proteins [52].

Following the selection of a recognition module type based on the target biomarker, strategies for signal transduction must be considered. The transducer module transfers molecular recognition events into physically detectable signals, such as colour, fluorescence, electrochemical signals, surface-enhanced Raman scattering (SERS), and surface plasmon resonance (SPR) changes [53]. The fluorescence method is simple, non-destructive, and of diverse applicability, making it suitable for portable POC devices [13]. Electrochemical biosensing is based on the electrochemical signal readout in target detection, while SPR is based on the resonant oscillation of conduction electrons at the interface between positive and negative permittivity materials when stimulated with incident light. Both electrochemical biosensing and SPR enable the real-time monitoring of analytes [12,31]. Electrochemical biosensing, when coupled with an aptamer recognition module, is a particularly attractive approach to the continuous in vivo detection of biomarkers [54].

### 2.2. Methods for Immobilisation of Recognition Modules

The recognition module, a critical component of an N-biosensor, needs to be immobilised on the surface of the functionalised deployable substrate. Depending on the orientation of the recognition module, the surface immobilisation approach can be classified into two types: random immobilisation [13] and oriented immobilisation [44]. Random immobilisation can be achieved via physical adsorption [33], an electrospray [55], amino coupling [31], or hydroxyl coupling [11]. Physical adsorption, based on hydrophobic interactions, electrostatic attraction, and ionic interactions [33], can result in random immobilisation for all types of recognition modules. Electrospray immobilisation applies an electric field to create microdroplets of recognition modules, which are uniformly deposited onto a substrate surface and become immobilised via physical absorption or chemical binding, depending on the substrate material [55]. Additionally, random immobilisation can be realised using amino- or hydroxyl-coupling approaches to protein/peptide and microorganism-based recognition domains [11,31], as these possess sufficient amino and hydroxyl groups on their surfaces, making them good targets for random immobilisation.

However, steric hindrance during random immobilisation can reduce the recognition ability of the module [8]. Oriented immobilisation, which utilises specific functional groups or binding sites on the recognition domain surface, provides an advanced approach [34]. This may be achieved through amino coupling, hydroxyl coupling, biotin coupling, thiol coupling, and biotag coupling.

Amino-coupling and hydroxyl-coupling methods were the first to be applied for the oriented immobilisation of recognition modules [42]. Unlike protein/peptide and microorganism-type recognition domains, DNA/RNA or small molecule-type recognition domains have limited (usually one or two) amino or hydroxyl groups, making them ideal for oriented immobilisation [42].

An alternative approach to oriented immobilisation is biotin coupling, applicable to antibody [8], aptamer [34], and antimicrobial peptide-based [49] recognition modules. This method involves immobilising streptavidin on the substrate surface, followed by the oriented immobilisation of a biotinylated recognition module [8]. The reverse approach uses a biotin-modified substrate to interact with a streptavidin-modified recognition domain [34]. Both methods are effective for biotin coupling.

Furthermore, thiol coupling can be utilised for the oriented immobilisation of protein or DNA type-recognition modules. The thiol groups, which originate from cysteine, glutathione-S-transferase (GST), and disulphide bridges in proteins [44], typically exhibit a limited and narrowly distributed presence on protein-type recognition modules, making them ideal for oriented immobilisation [56]. The thiol group can also be synthesised on one end of DNA/RNA-type recognition domains only [57,58], leading to oriented immobilisation. Following the modification of the recognition module with the thiol group, immobilisation is achieved through thiol coupling, forming a disulphide bond [44]. Importantly, the activation of the thiol group relies upon the presence of a sulfhydryl reductant (e.g., TCEP) [57,58]. An alternative approach to immobilising the thiol group-modified recognition module involves using gold as the functional material, as interactions between the thiol groups and gold result in immobilisation via sulphur–gold bond formation [59]. For example, light-based oriented immobilisation can be performed by applying femtosecond ultraviolet pulses to protein-recognition modules. The energy absorbed via tryptophan residues can break disulphide bonds and introduce reactive thiol groups that bind to gold electrode surfaces [60]. Consideration must be given to the equipment cost, light-source parameters for controlled exposure, and compatibility with the substrate material to avoid compromising the binding ability, but this immobilisation technique is safer than chemical methods, as it avoids the use of toxic reagents.

However, it is challenging to achieve controllable oriented immobilisation for protein-based recognition modules using existing coupling methods. A novel approach has, accordingly, been developed using biotag coupling [61], which provides natural binding tags as immobilisation sites for the oriented immobilisation of recombinant proteins. For instance, the Hexahistidine (*His*) tag has been widely used for protein immobilisation since it allows a stable and non-covalent interaction between pairs of histidine residues and a divalent metal ion such as nickel (Ni^2+^), which is anchored to the surface of a supporting substrate through a chelator such as nitrilotriacetic acid [61]. An alternative binding protein for oriented immobilisation is elastin-like protein (ELP), which has been integrated as part of a fusion protein to provide a hydrophobic binding point for the oriented immobilisation of the recognition module.

Collectively, the above-oriented immobilisation approaches are suitable for recognition modules based on proteins/peptides, DNA/RNA, small molecules, and bioreceptors. However, polymer-type recognition modules, including MIP and corona-phase polymers [39], are exceptions. These polymer-recognition modules are synthesised directly onto the surface of the supporting substrate, leading to difficulties in specifying orientation. The MIP approach has been used to recognise small molecules [39,62] and small proteins [30,63], but it still faces challenges with oriented immobilisation due to the randomly distributed templates in synthetic MIPs [30]. For the oriented immobilisation of MIPs to be achieved, it is crucial to first immobilise the templates in an oriented manner on the substrate surface before performing MIP synthesis. This strategy has been utilised in both surface MIP and epitope MIP to synthesise oriented MIP, while solid MIP is typically used for random immobilisation. An alternative recognition module, the corona-phase polymer, has been developed to recognise diverse biomarker targets [64,65], such as insulin, riboflavin, L-thyroxine, and oestradiol. The corona-phase polymer relies on the recognition capability of a polymer chain for capturing biomarkers, similar to aptamers but exhibiting superior chemical and enzymatic stability. The physical adsorption of the corona-phase polymer on the substrate is the most common approach to the immobilisation of the recognition module [64]. The oriented immobilisation of corona-phase polymers may thus be achieved via the attachment of a single end of the polymer chain to the supporting substrate, similar to the approach used for aptamers.

## 3. The In Vitro Application of N-Biosensors for Diagnoses in Clinical Specimens

Conventional plate-based detection approaches involve placing all clinical samples directly into a detection system. In contrast, N-biosensors developed on deployable substrates offer the unique capability to conduct the detection of target biomarkers from complex clinical biospecimens such as blood, plasma, serum, stool, urine, saliva, and tumour tissues (Table 2). Firstly, blood and blood derivatives (serum and plasma) constitute the most frequently used clinical samples for analysing various biomarkers, such as complete blood count (CBC), blood chemistry, specific proteins or antibodies, and genetic markers. Blood tests provide critical insight into various conditions, including anaemia, infection, and diabetes. Secondly, saliva samples are ideal candidates for diagnosing diseases of the stomatognathic system and heart disease. The collection of saliva samples is predominantly based on passive drool and swab collection methods. Thirdly, urine samples are routinely collected in clinical practice to diagnose urinary-system diseases and systemic disorders. Finally, stool samples provide ideal candidates for the diagnosis of bowel disease.

To date, diverse types of N-biosensors have found preclinical applications in the sensitive in vitro detection of various biomarkers within complex clinical samples, including small proteins [5,71], small molecules [68], DNA [58], microorganisms [6], and cells [52]. Examples of portable and POC N-biosensor approaches include SPR biosensing [5,67], microfluidic systems [66], electrochemical biosensing [56], and paper-based devices [72]. N-biosensors have demonstrated exceptional sensitivity by directly capturing target proteins from clinical samples without the need for complex pre-treatment of the samples. For instance, detecting various gene targets from complex clinical samples is necessary for diagnosing genetic disorders. Conventional plate-based approaches, such as polymerase chain reaction (PCR), require intricate and often time-consuming gene purification processes. In contrast, N-biosensors can streamline the gene-target capture process by eliminating the need for purification. This approach has successfully been applied to the measurement of nucleic acids in serum for the detection of Hepatitis B virus genes [58] and the diagnosis of cystic fibrosis [4,73].

Lastly, microorganisms in saliva and stool samples have been reported as valuable biomarkers for the diagnosis of various diseases. Again, PCR approaches to microorganism detection require complex gene purification processes. N-biosensing offers a straightforward method for detecting pathogenic microorganisms, with the surface epitope proteins and internal nucleic acids serving as potential targeting sites for these biosensors [6,7]. For instance, a capillary electrophoresis-based N-biosensor has been developed to recognise surface proteins in measuring *S. aureus* in stool, facilitating gastroenteritis diagnosis [69]. Furthermore, an advanced microfluidic system-based N-biosensor has been designed to detect *E. coli*, hPIC-3, and varicella–zoster viruses in saliva, again using surface protein recognition [6]. DNA microarray-based N-biosensing for the recognition of target nucleic acids has also been developed to detect bacterial pathogens within stool samples, assisting in the diagnosis of diarrheal disease [7]. Finally, a deployable N-biosensor has also been used to detect cancer cells similar in structure to a target microorganism from tumour tissue [52] and blood [70].

## 4. In Vivo Applications of N-Biosensing

In addition to testing in vitro, N-biosensing also provides the capability for in vivo detection without the need for sampling biological fluids or tissues. It offers distinct advantages, such as high surface-area-to-volume ratios and effective skin penetration for direct contact with analytes, enabling real-time biomarker detection with high sensitivity and minimum invasiveness. These features make N-biosensors particularly suitable for precise, localised measurements in PoC and continuous in vivo applications. As shown in Table 3, in vivo detection has been accomplished using a variety of N-biosensor platforms, including optical fibre, stainless steel, and needle-shaped microelectrodes.

Optical fibre-based fluorescent biosensing has found preclinical application in cytokine IL-1β detection within discrete regions of the brain [13] and spinal cord [8] of rats, assisting with the diagnosis of neuroinflammatory diseases and spinal cord injury. Additionally, an optical fibre-based SPR N-biosensor has been applied to the direct measurement of cytokeratin-17 from lung tissue, aiding lung cancer diagnosis [12].

Stainless steel represents a mechanically robust substrate for the benchtop development of N-biosensors for in vivo applications. Stainless steel microneedle-based SERS assays have demonstrated the capability for in vivo detection of glucose [10]. SERS assays using acupuncture needles have also been applied in detecting nitric oxide from fascia, brain, and muscle [44]. In addition to SERS assays, antibody-modified fluorescent stainless steel probes have been implemented in cytokine IL-1β measurement within the rat intrathecal space for the diagnosis of spinal cord injury [9].

Collectively, needle-shaped electrodes provide promising substrates for the development of electrochemical biosensors. The immobilisation of various recognition modules (e.g., antibodies, aptamers, and enzymes) on the surface of microelectrodes in N-biosensors has been widely reported in the literature, enabling a broad spectrum of in vivo applications. These include cytokine IFN-γ detection from rats’ subcutaneous pockets [34], cytokine TNF-α detection from skin phantoms [33], cytokine IL-6 detection from the rat brain [31], and glucose and lactate detection from the cerebral cortex [38]. A key point to note is that the above-mentioned sensors require removal from the body to conduct discrete measurements.

A continuous, in vivo N-biosensor offers the benefit of the real-time detection and analysis of biomarkers within living organisms; i.e., the sensor is implanted, and measurements are conducted whilst it is implanted. Continuous in vivo N-biosensors find application in patient-condition monitoring, particularly in tracking chronic disease and achieving timely disease management. These sensors may be designed with various configurations and degrees of invasiveness to adapt to the target biomarker and biofluid. Applications range from the minimally invasive intradermal POC assessment of metabolites such as glucose, lactate, and hydrogen peroxide within interstitial fluids using microelectrode arrays [76,77], to fully implantable N-biosensors for monitoring blood glucose levels [78]. Moreover, some N-biosensors are specifically designed for in vivo drug monitoring, enabling the precise assessment of pharmacokinetics and therapeutic efficacy, thus informing adjustments in medication selection and dosage [79]. For instance, an electrochemical N-biosensor has been developed specifically for monitoring levodopa levels, helping guide clinicians in the treatment of Parkinson’s disease [80]. Another example involves a drug-monitoring N-biosensor for phenoxymethylpenicillin, allowing for personalised treatment approaches. This in vivo application has the potential for further integration with closed-loop drug delivery systems for optimising antibiotic treatment dosage [22].

Continuous glucose monitoring (CGM) is a major application area for continuous, in vivo N-biosensors, with several FDA-approved subcutaneous CGM devices incorporating microneedle sensors inserted under the skin of the upper arm and abdomen [81,82]. These devices have demonstrated high-performance accuracy, effectively alerting users to both hypoglycaemic and hyperglycaemic events. Dexcom G7, for instance, has an overall mean absolute relative difference (MARD, the discrepancy between a CGM reading and a reference value from the YSI 2300 glucose analyser) of about 8.2% for sensors placed on the arm, with slight variations over the 10-day usage period [81]. Another CGM device, the Guardian™ Sensor, can be used with an automated insulin-pumping system for more convenient glucose management. It offers comparable biosensing performance with a slightly shorter sensor life of 7 days and a higher MARD of 8.7 ± 8.0% for an arm-placed sensor [82]. In comparison with these transcutaneous N-biosensors, the Eversense CGM system features fully implantable sensors placed in the upper arm. It can sustain up to 180 days of measurements, which significantly reduces the frequency of sensor replacements and the risks of sensor dislodgment. However, the Eversense sensor requires minor surgical procedures for insertion and removal, and it has shown MARDs of 9.1% for primary sensors and 8.5% for modified sensors, indicating slightly less accurate glucose detection [83]. While traditional implantable sensors may provide more stable tissue interlock and direct contact with a larger sample volume, eliminating the need for correlation and reducing repetitive interventions, especially for chronic disease management, N-biosensors show promise for integration into wearable devices with superior on-chip biosensing performance and potential to displace implantable sensors.

Despite the advancements in sensing, significant challenges persist in maintaining sensor sensitivity, long-term stability, and biocompatibility within complex in vivo environments. These engineering design issues pose considerable obstacles to the successful translation of continuous N-biosensors into clinical practice. Additionally, the development of tailored hardware and system designs is crucial for supporting real-time data transmission and analysis. Microminiaturisation is also essential for enabling painless insertion and the seamless integration of continuous N-biosensors into patients’ daily activities. Other biosensor types, such as wire-shaped biosensors, typically offer higher flexibility for easier integration with wearable applications, but they can exacerbate issues related to sensor-tissue interlocking and insertion. Alternatively, nanosensors provide better miniaturisation, but they are limited due to sensor stability, reproducibility, and system integration challenges because of the complexity and cost of fabrication at a nanoscale level [84]. Nonetheless, in vivo N-biosensors represent a promising tool for disease diagnosis and monitoring, offering significant potential improvements in clinical outcomes.

## 5. Conclusions and Future Prospects

The N-biosensor serves as a versatile platform that can be combined with various technologies to address a wide range of applications. It offers multiple advantages over conventional plate-based methods or nanobead-based methods. The deployable capability of N-biosensing allows for the direct capture of target biomarkers from complex clinical samples without the need for complicated sample collection or tedious processing procedures. Furthermore, N-biosensing enables the minimally invasive detection of disease biomarkers in vitro and in vivo for precision diagnosis. Considering its broad spectrum of applications, ongoing research aimed at further enhancing and adapting N-biosensor technology continues to be a promising field.

One primary future direction is to develop more sensitive and multiplexed N-biosensors for POC testing. To enhance the sensitivity of N-biosensing, novel signal amplification approaches are needed. For example, N-biosensors involving Clustered Regularly Interspaced Short Palindromic Repeats (CRISPR-Cas) technology show exceptional signal amplification capabilities due to their highly efficient trans-cleavage ability. This property holds significant promise for improving the sensitivity of N-biosensors in general [85]. For instance, integrating CRISPR-Cas technology with an N-biosensor has led to the development of a fibre-based ultrasensitive biosensing platform (1 fg/mL) capable of monitoring cytokines from whole blood, saliva, and sweat [45]. To develop a multiplexed N-biosensor capable of detecting multiple target biomarkers simultaneously, an effective spatial separation approach has been introduced [38,71]. This approach has been applied to a microneedle-based biosensor for the concurrent in vivo detection of glucose and lactate from the cerebral cortex [38].

Another long-term goal is to develop a real-time, stable, intelligent N-biosensor capable of continuous in vivo monitoring. To achieve the real-time biosensing of small molecules, the relevant functional enzyme acting as a recognition module must be suitable for the continuous real-time monitoring of signals, such as glucose oxidase [37]. The real-time biosensing of small proteins requires a structure-reversible recognition module containing a structure-reversible aptamer [86] and a structure-reversible antibody [87]. However, these are not the only challenges. For continuous in vivo detection to be achieved, sensors also require protection using anti-biofouling approaches to withstand long-term implantation. The materials used for such implantable sensors will also need to be biocompatible, and the sensors must be sterilisable. Additionally, methods for calibrating sensors in vivo and preventing sensor degradation or drift will require significant further research.

While these challenges have yet to be fully resolved, the successful implementation of real-time N-biosensors would enable several unique approaches to disease management. For example, one technological progression from a real-time N-biosensor is its integration with controllable therapeutic approaches to form a closed-loop, patient-tailored diagnosis–treatment system. In such a hypothetical system, the N-biosensor would act as a diagnostic module for the continual monitoring of biomarker levels. Following the signal processing of biomarker information, a command would feed back to the therapeutic module to perform a real-time intervention. This could involve the precise release of medicine [88] or targeted neural stimulation, such as vagal nerve stimulation, to manage inflammatory bowel disease, thus closing the feedback loop [89]. Another example is the application of closed-loop therapy for diabetes management [90], in which the biosensor monitors glucose levels and a controlled micro insulin pump provides personalised treatment. The paradigm shift towards personalised medicine has, thus, generated significant demand for closed-loop systems that enable real-time diagnoses and patient-specific treatment of chronic diseases. Considering the capabilities of N-biosensors for the in vivo monitoring of biomarkers, this technology is well suited to serving as the diagnostic component within such a closed-loop system.

## Figures and Tables

**Table 1:** Design specifications of N-biosensors that have been used to test various clinical analytes for the detection of specific biomarkers.

Signal Transducer	Supporting Substrate	Functional Material	Recognition Module	Immobilisation Method	Detected Biomarker
Fluorescence signal	Optical fibre	Gold nanoparticle	Antibody	N-(3-Dimethylaminopropyl)-N′-ethylcarbodiimide(EDC)/N-Hydroxysuccinimide (NHS)	IL-1β [13]
	Optical fibre	Streptavidin	Antibody	Biotin/streptavidin	IL-1β [8]
	Optical fibre	Streptavidin	Aptamer	Biotin/streptavidin	IFN-γ [29]
	Stainless steel	Poly(ethylene glycol) methyl ether methacrylate (PEG-MA)	Antibody	1,1′-Carbonyldiimidazole(CDI)	IL-1β [11]
	Stainless steel	Polydopamine	Molecularly imprinted polymer (MIPs)	Synthesis	IL-1β [30]
Electrochemical signal	Gold electrode	Graphene oxide	Antibody	EDC/NHS	IL-6 [31]
	Silicon microelectrode	Gold disc	Antibody	Sulfosuccinimidyl 6-[3′-(2-pyridyldithio) propionamido] hexanoate (sulfo-LC-SPDP)	IL-6 [32]
	Silicon microelectrode	Gold film	Antibody	Physical adsorption	TNF-α [33]
	Glassy carbon electrode	Streptavidin	Aptamer	Biotin/streptavidin	IFN-γ [34]
	Glassy carbon electrode	Gold nanoparticle	Glucose receptor	Cysteine	Glucose [35]
	Stainless steel microneedle	Platinum black	-	-	Glucose [36]
	Polymer needle	Gold nanoparticles	Glucose oxidase	Electrical adsorption	Glucose [37]
	Silicon microneedle	Platinum layer	Glucose oxidase and lactate oxidase	Glutaraldehyde	Glucose and lactate [38]
	Platinum microelectrode	Polypyrrole	MIP	Synthesis	Dopamine [39]
	Pencil graphite electrode	-	-	-	Propofol [40]
	Graphite electrode	Chromium (III) hexacyanoferrate	Oxalate oxidase	Glutaraldehyde	Oxalate [41]
	Graphite electrode	Carboxymethylated dextran film	DNA	EDC/NHS	cDNA [42]
	Hypodermic needle	Photoresist	-	Spray coating	Thyroid cancer tissue [43]
SPR	Optical fibre	Gold coating	Antibody	EDC/NHS	Cytokeratin 17 (CK17) [12]
SERS	Acupuncture needle	Gold nanoshells	3,4-diaminobenzene-thiol	Thiol	Nitric oxide [44]
	Microneedle	Silver layer	-	-	Glucose [10]

SPR, surface plasmon resonance; SERS, surface-enhanced Raman spectroscopy. Interleukin (IL), interferon (IFN), and tumour necrosis factor (TNF) are classes of cytokines.

**Table 2:** The in vitro application of N-biosensors for sensitive diagnoses in complex clinical samples.

Biomarker Type	N-Biosensor	Detection Range and Sensitivity	Clinical Sample	Biomarkers	Clinical Application
Small protein	Antibody-based SPR biosensor	Range: 0.33 × 10^9^–2.40 × 10^9^ RBC/mL LOD: 0.33 × 10^9^ RBC/mL	Blood	Blood group antigen [5]	-
	Antibody-based microfluidic system Glycosylphosphatidylinositol	Range: 40 pM–40 fM LOD: 10 fM	Serum	Prostate specific antigen [66]	Prostate disorders and cancer
	(GPI) bioreceptor based electrochemical biosensor	Range: 1.0–10.0 IU/mL LOD: 0.31 IU/mL	Serum	Anti-GPI IgG and IgM [56]	*Toxoplasma gondii*
	Aptamer-based SPR biosensor	Range: 0–100 ng/mL LOD: -	Stool	IpaH [67]	Shigellosis
Small molecule	Glucose biosensor	Range: 0.1–0.8 mM LOD: 0.1 mM	Serum	Glucose [68]	Diabetes
	Pencil graphite biosensor	Range: 30–240 µM LOD: 7.2 µM	Serum	Propofol [40]	Anaesthetic
	MIP-based SPR biosensor	Range: 20–1000 ng/mL LOD: 9.9 ng/mL	Plasma	Procalcitonin [62]	Sepsis
	Amperometric biosensor	Range: 2.5–100 μM LOD: 2.5 μM	Urine	Oxalate [41]	Renal stones
DNA	Electrochemical DNA sensor	Range: 8 × 10^−16^–5 × 10^−10^ mmol/L LOD: 2 × 10^−16^ mmol/L	Serum	DNA [4]	-
	Colourimetric DNA biosensor	Range: 0.5–100 nM LOD: 0.2 nM	Serum	DNA [58]	Hepatitis B virus (HBV) gene
Microorganism	Microfluidic system	Range of *E. coli*: 1 × 10–1 × 10^4^ cfu/mL;	Saliva	*E. coli*, hPIC-3, varicella-zoster Virus [6]	Pathogenic microorganism related disease
	DNA microarray	LOD: 10^3^ cfu/mL;	Stool	Bacterial pathogens [7]	Diarrhoea
	Capillary electrophoresis	LOD: 9.0 × 10^5^ cfu/mL;	Stool	*Staphylococcus aureus* [69]	Gastroenteritis
Cell	Aptamer assay	-	Tumour tissue	Cancer cells [52]	-
	Antibody assay	-	Blood	Lung circulating tumour cells [70]	Lung cancer

**Table 3:** In vivo applications of N-biosensing for sensitive diagnoses.

Signal Transducer	N-Biosensor	Detection Range and Sensitivity	In Vivo Detection Site	Biomarkers	Clinical Application
Fluorescence	Optical fibre-based antibody assay	Range: 3.9–500 pg/mL LOD: 1.2 pg/mL	Rat discrete brain regions	IL-1β [13]	Neuroinflammatory
	Optical fibre-based antibody assay	Range: 3.13–400 pg/mL LOD: 1.12 pg/mL	Rat spinal cord	IL-1β [8]	-
	Stainless steel-based antibody assay	Range: 12.5–200 pg/mL LOD: 3.2 pg/mL	Rat intrathecal space	IL-1β [9]	Spinal cord injury
Electrochemical	Aptasensor	Range: 10–500 pg/mL LOD: 10 pg/mL	Subcutaneous pockets	IFN-γ [34]	Inflammation
	Antibody biosensor	Range: 1–300 pg/mL LOD: 1 pg/mL	Rat brain	IL-6 [31]	-
	Antibody biosensor	Range: - LOD: 1 nM	Skin phantom	TNF-α [33]	-
	MIP-based biosensor	Range: 10–100 nM LOD: 4.5 nM	Striatum	Dopamine [39]	Parkinson’s Disease
	Oxidase biosensor	-	Cerebral cortex	Glucose and lactate [38]	Cerebral metabolism
SPR	Optical fibre-based biosensor	Range: 1 pg/mL–1 μg/mL LOD: 1 pg/mL	Lung	Cytokeratin 17 (CK17) [12]	Lung cancer
SERS	Microneedle-based assay	Range: 0–150 nM LOD: -	Skin phantom	Glucose [10]	-
	Acupuncture needle-based assay	Range: 0–100 μM LOD: 1 nM	Fascia, brain, muscle	Nitric oxide [44]	Paracrine
HPLC	Stainless steel fibre-based MIP assay	Range: 2.01–100.91 μg/mL LOD: 0.01 μg/mL	Liver	Luteolin [74]	Metabolism
AFM	Nanoneedle-based antibody biosensor	-	NIH3T3 cells	Tubulin [75]	Cytoskeleton-associated diseases

## Data Availability

No new data were created or analysed in this study.
